# Bioprocess for Production, Characteristics, and Biotechnological Applications of Fungal Phytases

**DOI:** 10.3389/fmicb.2020.00188

**Published:** 2020-02-14

**Authors:** Kritsana Jatuwong, Nakarin Suwannarach, Jaturong Kumla, Watsana Penkhrue, Pattana Kakumyan, Saisamorn Lumyong

**Affiliations:** ^1^Department of Biology, Faculty of Science, Chiang Mai University, Chiang Mai, Thailand; ^2^Center of Excellence in Microbial Diversity and Sustainable Utilization, Faculty of Science, Chiang Mai University, Chiang Mai, Thailand; ^3^Ph.D. Degree Program in Applied Microbiology, Department of Biology, Faculty of Science, Chiang Mai University, Chiang Mai, Thailand; ^4^School of Preclinic, Institute of Science, Suranaree University of Technology, Nakhon Ratchasima, Thailand; ^5^School of Science, Mae Fah Luang University, Chiang Rai, Thailand; ^6^Academy of Science, The Royal Society of Thailand, Bangkok, Thailand

**Keywords:** phytase, phytase production, purification, genetic engineering, biotechnological applications

## Abstract

Phytases are a group of enzymes that hydrolyze the phospho-monoester bonds of phytates. Phytates are one of the major forms of phosphorus found in plant tissues. Fungi are mainly used for phytase production. The production of fungal phytases has been achieved under three different fermentation methods including solid-state, semi-solid-state, and submerged fermentation. Agricultural residues and other waste materials have been used as substrates for the evaluation of enzyme production in the fermentation process. Nutrients, physical conditions such as pH and temperature, and protease resistance are important factors for increasing phytase production. Fungal phytases are considered monomeric proteins and generally possess a molecular weight of between 14 and 353 kDa. Fungal phytases display a broad substrate specificity with optimal pH and temperature ranges between 1.3 and 8.0 and 37–67°C, respectively. The crystal structure of phytase has been studied in *Aspergillus*. Notably, thermostability engineering has been used to improve relevant enzyme properties. Furthermore, fungal phytases are widely used in food and animal feed additives to improve the efficiency of phosphorus intake and reduce the amount of phosphorus in the environment.

## Introduction

**FIGURE 1:**
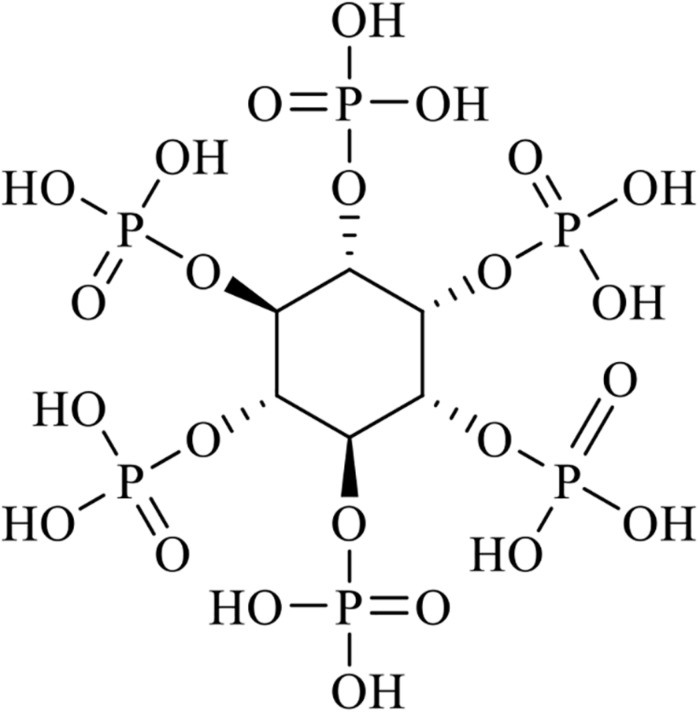
Structure of phytic acid (IP6, IUPAC).

Phytic acid is known as *myo*-inositol (1, 2, 3, 4, 5, 6) hexakisphosphate or phytate in salt form, as is shown in Figure 1. It is the major form of storage for phosphorus in plant tissues such as those in cereal grains, oilseeds, pollen, and legumes (Lott et al., 2000). Cereal grains and oilseed meals are major ingredients in animal feed as they are known sources of phosphorus, an essential macro-element required for animal growth (Selle and Ravindran, 2008). However, phosphorus in seeds exists predominately in the form of phytates (salt of phytic acid), and phytate phosphorus is not available to monogastric animals because they possess very low levels of phytase activity in their digestive tracts (Brinch-Pedersen et al., 2002; Vohra and Satyanarayana, 2003). Therefore, phosphate supplementation is required for optimal animal growth (Chen et al., 2008). However, a large amount of undigested phytate phosphorus is excreted along with animal waste and this is known to cause algal blooms and eutrophication in surface waters. Fungal phytases are widely produced in fermentation processes and are commonly used to overcome the nutritional and environmental problems caused by phytates. Currently, phytases are being utilized as a major animal feed additive (Mullaney et al., 2000).

**FIGURE 2:**
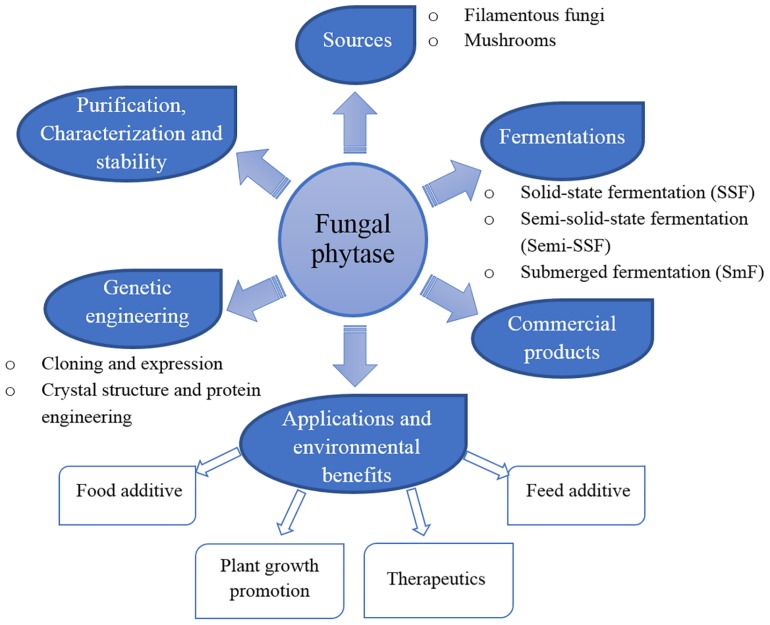
The summarization of fungal phytase.

Phytases (*myo*-inositol hexakisphosphate phosphohydrolases) are a class of phosphatases that catalyze the hydrolysis of phytates to *myo*-inositol, inositol phosphate, and inorganic phosphates (Wodzinski and Ullah, 1996; Wyss et al., 1998). Phytases were first identified by Suzuki et al. (1907) who found an enzyme present in rice bran. Moreover, phytases are widespread in nature and can be produced from various host sources including plants, animals, and microorganisms (Yao et al., 2011). Based on their catalytic function and structure, the first and most extensively studied group of phytases are classified as histidine acid phosphatases (HAPs) that have been isolated from filamentous fungi, bacteria, yeasts, and plants (Mullaney et al., 2000). Phytases have been commonly detected in many fungal species and are most often characterized by their presence in those fungal species (Mukhametzyanova et al., 2012; Singh and Satyanarayana, 2014). However, the physico-chemical characteristics and catalytic properties of phytases depend upon the different fungal strains that serve as their source. Thus, the phytase production of fungi is dependent upon differing optimum temperatures and pH values that range from neutral to acidic (pH 1–6) or alkaline (pH 8–14) (Yao et al., 2011; Singh and Satyanarayana, 2014). *Aspergillus* has been most commonly employed for phytase production. Thus, the first generation of commercially available fungal phytase obtained from *A. niger* was marketed in 1991 and has been applied for use in various industries ever since, such as in the production of human food and animal feed as well as in the preparation of *myo*-inositol phosphates. Furthermore, phytases have also been used in the semi-synthesis of peroxidase employed in the paper and pulp industries and as a soil amendment and plant growth promoter (Singh et al., 2011). Several fungal strains have been extensively studied for phytase production, purification, characterization and stability, cloning and expression. Consequently, their potential biotechnological applications have been reported (Yao et al., 2011; Lei et al., 2013). The summarization of phytases is shown in Figure 2. This review addresses the properties and potential biotechnological applications of fungal phytases.

## Sources and Production of Fungal Phytases

Phytases are produced in nature in a wide range of plant and animal tissues and microorganisms such as bacteria, yeast, and fungi (Vohra and Satyanarayana, 2003). Most scientific works have focused on microbial phytases, particularly those obtained from filamentous fungi such as *Aspergillus*, *Myceliophthora*, *Mucor*, *Penicillium*, *Rhizopus*, and *Trichoderma* (Tseng et al., 2000; Sabu et al., 2002; Roopesh et al., 2006; Dailin et al., 2019). *Aspergillus ficuum* NRRL 3135 has been defined as the most active fungal phytase producer and has most commonly been employed at the commercial level (Chelius and Wodzinski, 1994). Other filamentous fungal species that can produce phytase during the fermentation process are *A. carbonarius*, *A. fumigatus*, *A. niger*, *A. oryzae*, *Cladosporium* species, *Mucor piriformis*, and *Rhizopus oligosporus* (Howson and Davis, 1983; Casey and Walsh, 2004; Quan et al., 2004; Salmon et al., 2012). Moreover, the phytase activity of some edible mushrooms, such as *Agaricus bisporus*, *Agrocybe pediades*, *Ceriporia* sp., *Ganoderma stipitatum*, *Grifola frondosa*, *Lentinula edodes*, *Peniophora lycii*, *Pleurotus cornucopiae*, *Schizophyllum commune* and *Trametes pubescens*, has also been reported (Lassen et al., 2001; Collopy and Royse, 2004; da Luz et al., 2012).

The production of fungal phytases has been achieved using three different fermentation methods; namely solid-state (SSF), semi-solid, and submerged fermentation (SmF) (Han et al., 1987; Shivanna and Venkateswaran, 2014). Fungal phytases are commonly produced using solid-state fermentation (SSF) methods, in which agricultural waste and other cheap natural substrates are used as substrates in the SSF process (Sabu et al., 2002; Awad et al., 2014; Huang et al., 2018). Solid-state fermentation is defined as the fermentation process of microorganisms grown on a solid material surface with absence or near absence of free water. However, the process must have enough moisture content to support the growth of microorganisms. Solid-state fermentation of phytase production by fungi has been employed using agricultural waste and other cheap natural materials as substrates. This has been established due to the fact that these substrates can support fungal growth along with their natural metabolism (secreted enzymes). Importantly, fungi can grow on the solid substrate in the same way they typically grow in nature (Bhargav et al., 2008; Kumar and Kanwar, 2012). Furthermore, SSF involving fungi offers high volumetric productivity and high yields of enzyme production. In this method, enzymes can be easily extracted with water and the process is recognized as being less expensive, easier to use and less time-consuming (Bhargav et al., 2008). The process has been widely used in the fermentation industry, particularly for enzyme production (Pandey et al., 1999; Soccol et al., 2017). Several studies of SSF have been performed using filamentous fungi for phytase production, such as *A. flavus*, *A. ficuum*, *A. niger*, *A. tubingensis*, *Ganoderma stipitatum*, *Grifola frondosa*, *M. racemosus*, *Penicillium purpurogenum*, *R. oligosporus*, *R. oryzae*, *S. commune*, *Thermomyces lanuginosus*, and *Trametes versicolor*. Some of the substrates generally used for phytase production are citrus peels, wheat bran, wheat straw, soybean meal, rice bran, oil cakes, corn cobs, corn bran, and coconut oil cakes. For example, Sabu et al. (2002) have reported on phytase production by *R. oligosporus* on coconut oil cake substrate in SSF. In a recent study, triticale waste was used as a substrate for the evaluation of phytase production by *A*. *niger* (Neira-Vielma et al., 2018). Additionally, phytase production was investigated by SSF using mixed substrates. Roopesh et al. (2006) reported on phytase production by *M. racemosus* using combinations of wheat bran and various oil cakes which gave the highest phytase activity with 32.2 U/gds. Then, phytase production by *Penicillium purpurogenum* was investigated by SSF using mixed substrates consisting of corn cob and corn bran (Awad et al., 2014). In a recent study, Kanti and Sudiana (2018) reported on the application of *Aspergillus niger*, *Neurospora sitophila*, and *R. oryzae* on mixed rice straw powder and soybean curd residues. *Neurospora sitophila* showed the highest level of phytase production at 195.66 U/g followed by *A. niger* and *R. oryzae*. However, not only SSF has been investigated for phytase production, but a number of research studies have also investigated phytase production involving SmF and semi-solid fermentation methods (Han et al., 1987; Salmon et al., 2016; Shah et al., 2017). The production of phytase from several fungal strains has been investigated during SmF. For example, phytase production from *Aspergillus fumigatus*, *A. japonicus*, *A. niger*, *Muscodor* sp., and *Ganoderma* sp. MR-56 was investigated under SmF conditions using wheat bran as a substrate (Mandviwala and Khire, 2000; Mohan et al., 2004; Alves et al., 2016; Maller et al., 2016; Salmon et al., 2016). Coban and Demirci (2014) have produced and optimized culture conditions for *A. ficuum* NRRL 3135 using phytase selective medium containing sodium phytate as a substrate. In addition, Kanti and Sudiana (2018) produced phytase under SmF using mixed rice straw power and soybean curd residue as a substrate and *Aspergillus niger*, *Neurospora sitophila*, and *R. oryzae* as fungal strains to increase phytase production. Maximum phytase activity was obtained by *N. sitophila* (195.66 U/g) at 96 h of incubation. Also, Coban and Demirci (2015a) studied phytase production by optimizing important nutrients using *A. ficuum* in SmF (glucose, Na-phytate, and CaSO_4_) and the effect of pH and temperature on phytase activity in bench-top bioreactors by conducting fed-batch fermentations. The results revealed that the optimum glucose, Na-phytate, and CaSO_4_ concentrations were 126, 14, and 1.1 g/L, respectively. Optimum pH and temperature values were 5.5 and 55°C for *A. ficuum* phytase activity. Therefore, these conditions indicate that phytase activity increased by improving the media and process conditions. Table 1 presents several examples of fungal phytase sources and a variety of different methods of fermentation that have been employed for phytase production. Various production factors, such as the type of strain, culture conditions, the nature of the substrate, and availability of the nutrients, are all considered critical factors that can affect yields. Consequently, each of those factors should be taken into consideration when fungal phytase production is undertaken (Pandey et al., 2001).

**TABLE 1:** Types of fermentation and substrates for fungal phytase production.

Fungal taxa	Fermentation type	Substrate	References
*Aspergillus* sp. FS3	SSF	Citric pulp	Spier et al., 2008
*Aspergillus* sp. F3	SSF	Citrus peel	Rodriguez-Fernandez et al., 2010, 2012, 2013
*Aspergillus flavus*	SSF	Wheat bran	Karthik et al., 2018
*Aspergillus japonicus*	SmF	Wheat bran	Maller et al., 2016
*Aspergillus ficuum*	Semi-SSF	Wheat straw	Han et al., 1987
*Aspergillus ficuum* NRRL 3135	SmF	Na-phytate	Coban and Demirci, 2014
*Aspergillus ficuum* SGA 01	SSF, SmF	Wheat bran	Shivanna and Venkateswaran, 2014
*Aspergillus ficuum*	SSF	Wheat straw	Shahryari et al., 2018
*Aspergillus fumigatus*	SmF	Wheat bran	Mohan et al., 2004
*Aspergillus niger*	SmF	Wheat bran	Mohan et al., 2004
*Aspergillus niger*	SSF, SmF	Mixed rice straw power and soybean curd residue	Kanti and Sudiana, 2018
*Aspergillus niger*	SmF	Chickpea flour	Shah et al., 2017
*Aspergillus niger*	SSF, SmF	Wheat bran	Papagianni et al., 1999, 2001
*Aspergillus niger*	SSF	Soybean meal	Saithi and Tongta, 2016
*Aspergillus niger* CFR 335	SSF, SmF	Wheat bran	Shivanna and Venkateswaran, 2014
*Aspergillus niger* NCIM 563	SSF, SmF	Wheat bran	Ebune et al., 1995; Mandviwala and Khire, 2000
*Aspergillus niger* NCIM 563	SmF	Rice bran	Bhavsar et al., 2008
*Aspergillus niger* NCIM 612	SSF	Rice bran	Das and Ghosh, 2014
*Aspergillus niger* 7A-1	SSF	Triticale	Neira-Vielma et al., 2018
*Aspergillus tubingensis*	SSF	Wheat bran	Qasim et al., 2017
*Ganoderma* sp. MR-56	SmF	Wheat bran	Salmon et al., 2016
*Ganoderma stipitatum*	SSF	Wheat bran	Spier et al., 2012
*Grifola frondosa*	SSF	Brown rice	Huang et al., 2018
*Mucor racemosus* NRRL 1994	SSF	Wheat bran and sesame oil cake	Roopesh et al., 2006
*Muscodor* sp.	SmF	Wheat bran	Alves et al., 2016
*Neurospora sitophila*	SSF, SmF	Mixed rice straw power and soybean curd residue	Kanti and Sudiana, 2018
*Penicillium purpurogenum* GE1	SSF	Corn cob and corn bran	Awad et al., 2014
*Rhizopus* spp.	SSF	Oilcakes	Ramachandran et al., 2005
*Rhizopus oligosporus*	SSF	Coconut oil cake	Sabu et al., 2002
*Rhizopus oligosporus* MTCC556	SmF	Wheat bran	Haritha and Sambasivarao, 2009
*Rhizopus oryzae*	SSF, SmF	Mixed rice straw power and soybean curd residue	Kanti and Sudiana, 2018
*Schizophyllum commune*	SSF	Wheat bran	Salmon et al., 2012
*Sporotrichum thermophile*	SmF	Wheat bran	Singh and Satyanarayana, 2008
*Thermoascus aurantiacus*	SmF	Wheat bran	Nampoothiri et al., 2004
*Thermomyces lanuginosus*	SSF	Wheat bran	Berikten and Kivanc, 2013
*Thermomyces lanuginosus*	SmF	Rice flour	Bujna et al., 2016
*Trametes versicolor*	SSF	Wheat bran	Spier et al., 2012

*SSF, solid-state fermentation; Semi-SSF, semi-solid-state fermentation; SmF, submerged fermentation.*

Phytase production using different fungal strains is affected by differing culture conditions (Qasim et al., 2017; Neira-Vielma et al., 2018). Optimum conditions for the production of phytases obtained from different fungal strains have been established by changing both nutrient and physical conditions. Additionally, various sources, such as glucose and sucrose, were used as a carbon source, while ammonium sulfate [(NH_4_)_2_SO_4_], ammonium nitrate (NH_4_NO_3_), yeast and malt extract were used as nitrogen sources for the fermentation process, as is shown in Table 2. To check the optimum physical conditions for phytase production, such as pH value and temperature, different ranges of the initial pH of the culture medium and different temperatures of incubation have been employed (Alves et al., 2016; Qasim et al., 2017). For an example, the production of phytase from *A. flavus* ITCC 6720 was investigated by SSF on mustard cake as a substrate. The Optimized conditions of production involved supplementation with 0.5% malt extract and glucose at 58% moisture level, 10% inoculum level, and inoculum age-72 h old. The maximum phytase activity of 34 to 112.25 U/g fermented substrate was produced on the 4^*th*^ day under an incubation temperature of 37°C and a pH value of 6.0 (Gaind and Singh, 2015), while *A. flavus* produced maximum phytase (80 U/g of solid substrate) in SSF using wheat bran as a solid substrate at a pH value of 6 after 7 days of the fermentation period at 30°C in the medium containing glucose (2%) as a carbon source and tryptone (1%) as a nitrogen source (Gull et al., 2013). Phytase production by filamentous mushrooms has also been studied. Salmon et al. (2012) reported that a maximal level of phytase (113.7 U/gds) was obtained in wheat bran-based medium involving 50% humidity with 7.5% of the biomass at 33°C and at a pH value of 7.0 over 72 h, which resulted in a 285% level of improvement in terms of the amounts of enzymes obtained. *Ganoderma applanatum* synthesized phytase in a medium comprised of soybean molasses as a carbon source and yeast extract as a nitrogen source at 30°C at 150 rpm, a pH of 6.0 and a 3% inoculum rate through SmF (Salmon et al., 2016). The production of phytase by fungal strains has been observed at a wide range of initial pH values and temperatures ranging from 4.5–8.0 and 27–50°C, respectively. Various culture conditions used for fungal phytase production by filamentous fungi are presented in Table 2. However, the cultivation of a filamentous fungus is often accompanied by several challenges, such as clumpy growth, high broth viscosity, insufficient oxygen, and mass transfer which results in reduced levels of productivity. Therefore, in order to improve biomass and product formation during cultivation of filamentous microorganisms, the process was performed under microparticle-enhanced cultivation (MPEC). To date, microparticle talc powder (magnesium silicate), aluminum oxide, and titanium oxide have been used in several studies to increase the production of enzymes in the fermentation of filamentous fungi such as *A. ficuum*, *A. niger*, *A. oryzae*, *A. sojae*, *Caldariomyces fumago*, *Cerrena unicolor*, and *Pleurotus sapidus* (Singh, 2018). Kaup et al. (2008) studied the effects of the different microparticle sizes of talc or aluminum microplates on chloroperoxidase (CPO) formation by *Caldariomyces fumago*. They observed that small particles (≤42 μm diameter) dispersed *C. fumago* to singer hypha, while particles around 500 μm diameter did not make any difference in the growth morphology or CPO formation productivity by *C. fumago*. Driouch et al. (2012) studied *A. niger* fermentation in submerged culture by adding titanate microparticles (TiSiO_4_, 8 μm) to the growth medium. They reported that fructofuranosidase and glucoamylase productions were increased by 3.7-fold to 150 U/mL and 9.5-fold to 190 U/mL, respectively when an additional 25 g/L of TiSiO_4_ was added to the fermentation medium as compared to the control. Driouch et al. (2011) also reported that fructofuranosidase production was increased by 3.5-fold in the presence of microparticles of either 10 g/L of talcum or 20 g/L aluminum oxide using *A. niger* in the fermentation medium. Additionally, Coban et al. (2015b, c) studied the effects of different microparticles on *A. ficuum* phytase production. They reported that *A. ficuum* phytase production was increased and the fungal pellet size was decreased after the addition of microparticles to the batch fermentation. Therefore, the use of a novel method of MPEC could be applied for the purposes of improved biomass and product formation of hydrolytic enzymes during cultivation of filamentous fungi.

**TABLE 2:** Culture conditions for phytase production by filamentous fungi.

Fungal taxa	Carbon source	Nitrogen source	^pH^opt	^T^opt (°C)	References
*Aspergillus heteromorphus*	Glucose	(NH_4_)_2_SO_4_	6.0	30	Lata et al., 2013
*Aspergillus ficuum* PTCC 5288	Glucose	(NH_4_)_2_SO_4_	−	35	Jafari-Tapeh et al., 2012
*Aspergillus ficuum* SGA 01	−	−	4.5	30	Shivanna and Venkateswaran, 2014
*Aspergillus flavus*	Glucose	Tryptone	6.0	30	Gull et al., 2013
*Aspergillus flavus* ITCC 6720	Glucose	Malt extract	6.0	37	Gaind and Singh, 2015
*Aspergillus fumigatus*	Wheat bran	Yeast extract	5.5	55	Mohan et al., 2004
*Aspergillus niger*	Wheat bran	Yeast extract	5.5	55	Mohan et al., 2004
*Aspergillus niger*	Wheat bran	Peptone	5.5–5.8	30	Papagianni et al., 1999
*Aspergillus niger*	Glucose	Tryptone	5.0	30	Sandhya et al., 2015
*Aspergillus niger* van *Tieghem*	Glucose and starch	NH_4_NO_3_	6.5	30	Vats and Banerjee, 2002
*Aspergillus niger* CFR 335	−	−	4.5	30	Shivanna and Venkateswaran, 2014
*Aspergillus niger* NCIM 563	Cowpea meal	−	−	37	Mandviwala and Khire, 2000
*Aspergillus niger* NCIM 563	Glucose	NaNO_3_	5.5	30	Bhavsar et al., 2008
*Aspergillus niger* NRF9	Wheat bran	NaNO_3_	4.5	30	Gupta et al., 2014
*Aspergillus ficuum* NRRL 3135	Glucose	NaNO_3_	4.5	33	Coban and Demirci, 2014
*Aspergillus tubingensis* SKA	Glucose	(NH_4_)_2_SO_4_	5.0	30	Qasim et al., 2017
*Ganoderma applanatum*	Soybean molasses	Yeast extract	6.0	30	Salmon et al., 2016
*Penicillium purpurogenum* GE1	Glucose	Peptone	8.0	27	Awad et al., 2014
*Rhizopus oligosporus* MTCC556	Glucose	Peptone	6.0	−	Haritha and Sambasivarao, 2009
*Rhizomucor pusillus*	Glucose	NH_4_NO_3_	6.0	50	Chadha et al., 2004
*Schizophyllum* sp.	Sucrose	Yeast extract	7.0	30	Salmon et al., 2011
*Schizophyllum commune*	Sucrose	Yeast extract	7.0	33	Salmon et al., 2012
*Sporotrichum thermophile*	Starch	Peptone	5.0	45	Singh and Satyanarayana, 2008
*Thermoascus aurantiacus*	Glucose and starch	Peptone	5.5	55	Nampoothiri et al., 2004
*Thermomyces lanuginosus*	Wheat bran	NaNO_3_	5.5	45	Gulati et al., 2007
*Thermomyces lanuginosus*	Rice flour	NaNO_3_	−	47	Bujna et al., 2016

## Purification and Characterization of Fungal Phytases

The purification of enzymes is necessary in order to study their biochemical properties as well as to understand their structural and functional relationships. Various methods have been used to purify relatively large numbers of protein molecules, while separation is often affected by the differences of the target protein and the properties of other substances present in the sample, such as solubility, precipitation, size, polarity, and the binding affinity of ammonium sulfate/acetone/ethanol precipitation followed by ultrafiltration, ion exchange, and gel filtration chromatography (Vohra and Satyanarayana, 2003; Boyce and Walsh, 2007). Therefore, combinations of two or more methods are commonly used for the purification of fungal phytases (Zhang et al., 2013a; Neira-Vielma et al., 2018). Fungal phytases belong to a class of HAPs. Most fungal phytases are active under acidic pH conditions in the optimum pH range of 2.0–6.0 (Vohra and Satyanarayana, 2003; Singh et al., 2011; Yao et al., 2011; Zhang et al., 2013b), but some fungal phytases, for instance, *Agaricus biosporus* and *Rhizopus microsporus var. microsporus*, belong to 5.0–8.0 and 9.5 pH, respectively (Collopy and Royse, 2004; de Oliveira Ornela and Guimaraes, 2019). However, the thermostability of phytases is also essential for their use in animal feed (Dersjant-Li et al., 2015). Fungal phytases are active in the optimum temperature range of 37–67°C. Various studies have reported that different fungal phytases are active under different optimal conditions with regard to pH and temperature (Lassen et al., 2001; Zhu et al., 2011; Neira-Vielma et al., 2018). Fungal phytase obtained from *A. niger* van *Tieghem* showed a high degree of specific activity under an optimal temperature range of 52–55°C together with an optimal pH value of 2.5 (Vats and Banerjee, 2005). Moreover, only a few studies have confirmed that fungal phytases are wide molecular mass proteins ranging from 14 to 353 kDa depending on the different fungal strains (Collopy and Royse, 2004; Sariyska et al., 2005). The purification steps and biochemical properties of fungal phytases are presented in Table 3.

**TABLE 3:** Purification steps and biochemical properties of fungal phytases.

Fungal taxa	Steps in purification	Specific activity	MW (kDa)	^pH^opt	^T^opt (°C)	Km (μM)	References
*Agaricus bisporus*	Anion-exchange, ultrafiltration and gel filtration	14.7 U/mg	14	5.0–8.0	>60	−	Collopy and Royse, 2004
*Agrocybe pediades*	Ultrafiltration, anion exchange	400 U/mg	59	5.0–6.0	50	−	Lassen et al., 2001
*Aspergillus foetidus*	Ammonium sulfate precipitation, gel filtration	12.6 FTU/mg	129.6	5.5	37	−	Ajith et al., 2019
*Aspergillus ficuum* AS3.324	Ammonium sulfate fraction and anion exchange	−	68.5	2.0, 5.5	50	750	Zhang et al., 2003
*Aspergillus ficuum* NTG-23	Ion-exchange and gel filtration	150.1 U/mg	65.5	1.3	67	295	Zhang et al., 2010
*Aspergillus ficuum*	Ion exchange	178.76 U/mg	67.5–81.6	5.0	58	0.124	Ullah et al., 2003
*Aspergillus flavus* ITCC 6720	Acetone precipitation, ion exchange and ultrafiltration	46.53 U/mg	30	7.0	45	−	Gaind and Singh, 2015
*Aspergillus fumigatus*	Ammonium sulfate precipitation, anion exchanger, and gel filtration	0.23 U/mg	118	6.0	40	7200	Sanni et al., 2019
*Aspergillus niger* var. *Tieghem*	Ion-exchange and gel filtration	22,592 U/mg	353	2.5	52–55	0.606	Vats and Banerjee, 2005
*Aspergillus niger* ATCC 9142	Ultrafiltration, ion exchange, gel filtration, and chromatofocusing	89.6 U/mg	84	5.0	65	100	Casey and Walsh, 2003
*Aspergillus niger* 307	Ultrafiltration, gel filtration, and anion-exchange	339.72 U/mg	39	2.62, 5.05	55–58	0.929	Sariyska et al., 2005
*Aspergillus niger* CFR 335	Ammonium sulfate fractionation, dialysis, and anion-exchange	32.6 ± 3.1 U/mg	66	4.5	30	80 ± 0.1	Gunashree and Venkateswaran, 2008
*Aspergillus niger* 7A-1	Ultrafiltration and ion exchange	8.38 U/mg	89	5.3	56	220	Neira-Vielma et al., 2018
*Aspergillus oryzae*	Anion exchange and ion exchange	2 U/ml	74	5.5–6.0	50	−	Uchida et al., 2006
*Ceriporia* sp.	Ultrafiltration, anion exchange	700 ± 80 U/mg	59	5.5–6.0	55–60	−	Lassen et al., 2001
*Cladosporium* sp. FP-1	Ion exchange and gel filtration	909 U/mg	32.6	3.5	40	15.2±3.1	Quan et al., 2004
*Flammulina velutipes*	Ion exchange and anion exchanger and blue	3.4 U/mg	14.8	5.0	45	−	Zhu et al., 2011
*Lentinus edodes*	Ion-exchange and anion exchange	3.11 U/mg	14	5.0	37	−	Zhang et al., 2013b
*Mucor hiemalis*	Ultrafiltration, diafiltration, ion exchange, gel filtration and hydrophobic interaction	46.7 U/mg	45	5.0–5.5	55	−	Boyce and Walsh, 2007
*Peniophora lycii*	Ultrafiltration and anion exchange	1080±110U/mg	72	4.0–5.0	50–55	−	Lassen et al., 2001
*Penicillium simplicissimum*	Ultrafiltration, cation exchange, anion-exchange and gel filtration	3245 U/mg	65	4.0	55	−	Tseng et al., 2000
*Rhizopus oligosporus*	Acetone fractionation, gel filtration and ion exchange	9.47 U/mg	−	4.5	55	150	Buckle, 1988
*Rhizopus microsporus* var. *microsporus*	Ion exchange and gel filtration	0.8 U/mg	55	9.5	65	413	de Oliveira Ornela and Guimaraes, 2019
*Schizophyllum commune*	Ion exchange and anion-exchange and gel filtration ultrafiltration, anion exchange	5260.5 U/mg	72.5	4.6	50	248	Zhang et al., 2013a
*Trametes pubescens*	Anion-exchange, ion exchange and blue gel	1210±30U/mg	62	5.0–5.5	50	−	Lassen et al., 2001
*Volvariella volvacea*	Ion exchange, blue gel and gel filtration	−	14.8	5.0	45	−	Xu et al., 2012

## Cloning and Expression of Fungal Phytases

Fungal phytases are widely used as a feed additive to increase phosphorus availability and reduce phosphorus excretion in manure (Brinch-Pedersen et al., 2002). However, wild filamentous fungal strains that produce enzymes can rarely meet the industrial demand. Genetic engineering strategies have been used to obtain recombinant strains that produce high levels of enzymes for industrial interests (Correa et al., 2015).

The use of phytase transgenic plants is one of the approaches that may help to mitigate the problems associated with phytate indigestibility. There are two possible strategies for altering phytate levels. One involves blocking the phytate biosynthetic pathway or degrading phytate in developing seeds. The other involves altering the steps of phytate biosynthesis, but this has the potential disadvantage of affecting many other cellular processes associated with inositol phosphates. Expressing phytase transgenes during seed development to modify the final composition of harvested seeds is an alternative development process (Chiera et al., 2004). Recently, heterologous expression of phytases in plants to produce plant seeds containing high phytase levels has received increasing amounts of attention. Previous publications have reported that if cereal grains or seeds contain enough phytase, the supplementation of microbial phytase additives in animal feed will not be required. Additionally, the transgenic plants would access phosphate from the soil that contains phytate-phosphate complexes (Reddy et al., 2017). Ideal phytase transgenic expression is based on the target application, such as with root expression and seed expression. Therefore, the selection of enzyme sources and physical properties with regard to pH stability and thermo-stability can affect the success of the expression. In acidic soil, phytases with a low isoelectric point (pI) are more effective in hydrolyzing phytates in the soil than phytases with high pI values. Notably, in basic soil, phytases with high pI values are preferable to phytases with low pI values. Researchers have made several attempts to reclaim sustainable phosphate utility, plant nutrition, and ecological balance in various studies. There have been many research studies involving phytase transgenic plants. Interestingly, a recombinant fungal phytase has been constructed in soybeans that have been widely used in livestock feed (Li et al., 1997). Chiera et al. (2004) and Li et al. (2009) found that phytase genes obtained from *A. ficuum* under the regulations of root specific Pyk10 promoters in soybeans and transgenic plants exhibited phytase activity. The development of soybeans containing low seed phytate levels would increase phosphorus availability and eliminate the need for phytase supplementation in animal feed or livestock. Moreover, Lucca et al. (2001) recorded the expression of *A. fumigatus* phytase in rice (*Oryza sativa*). Pen et al. (1993) reported that transgenic tobacco seeds express *A. niger* phytases. These results confirm that transgenic tobacco seeds expressed *A. niger* phytases and have beneficial effects on phosphate liberation while enhancing the broiler growth rate over commercially produced phytases. Notably, the expression of the *A. niger* phytase gene with an ER signal peptide into canola (*Brassica napus*) was recorded. The results indicate that this transgenic plant could accumulate phytase (Peng et al., 2006). Similar results were reported by Wang et al. (2013) who introduced the phytase gene into canola and it greatly boosted phosphorus uptake, plant biomass and seed yields in the presence of a phosphate source. Numerous research studies have proven that enhanced phytate-phosphate availability in soil can be achieved by expressing the phytase gene in transgenic plants, as is shown in Table 4. Some evidence has shown that plant phytase expression may somehow interfere with the refolding of the enzyme or may provide an environment that is not favorable for refolding, and this could affect the enzyme properties. Yoon et al. (2011) reported on the expression levels of six microbial phytases in *Chlamydomonas reinhardtii*, and concluded that the N-terminal signal peptide and codon optimization affected the degree of efficient expression. Constitutive and inducible mechanisms in plant seeds and microorganisms have been identified. The constitutive and germination-inducible mechanisms in plant seeds and pollen are involved in the regulation of phytate breakdown during germination. The activity of the hydrolytic enzymes and their rate of synthesis were controlled by these two main mechanisms (Greiner, 2007). Li et al. (1997) studied using the phytase gene obtained from *A. niger* inserted into soybean transformation plasmids under the control of constitutive (35S CaMV promoter) and seed specific promoters (β-conglycinin α’-subunit promoter), with and without a plant signal sequence. They reported that phytase activity was detected in the culture medium obtained from transformants that received constructs containing the plant signal sequence, and this confirmed the expectation that the protein would follow the default secretory pathway. Therefore, the recombinant phytase values obtained from their studies suggested that the additional protein stability would be required to withstand the elevated temperatures involved in soybean growth processing.

**TABLE 4:** Transgenic phytase in fungi.

Gene source	Recombinant plant	References
*Aspergillus ficuum*	Soybean, *Glycine max*	Li et al., 2009
*Aspergillus fumigatus*	Rice, *Oryza sativa*	Lucca et al., 2001
	Wheat, *Triticum aestivum*	Brinch-Pedersen et al., 2000
		Brinch-Pedersen et al., 2006
*Aspergillus japonicus*	Wheat *Triticum aestivum*	[Bibr B1][Bibr B100]
*Aspergillus neoniger*	Yeast, *Pichia pastoris*	Zhou et al., 2019
*Aspergillus niger*	Tobacco, *Nicotiana tabacum*	Pen et al., 1993
	Maize seed	Chen et al., 2008
	Soybean, *Glycine max*	Chiera et al., 2004
	Canola, *Brassica napus*	Wang et al., 2013
		Peng et al., 2006
	Tobacco, *Nicotiana tabacum*	Giles et al., 2018
	Algal, *Chlamydomonas reinhardtii*	Erpel et al., 2016
*Penicillium chrysogenum*	Fungi, *Penicillium griseoroseum*	Correa et al., 2015

In addition, the thermophilic mold *Sporotrichum thermophile* has been investigated in terms of the cloning and expression of phytase heterologously in bacteria (e.g., *Escherichia coli*) or yeast (e.g., *Pichia pastoris*) (Ranjan et al., 2015; Singh et al., 2018). The recombinant phytase (rSt-Phy) of the thermophilic mold *S. thermophile* (*St-Phy*) had been cloned and expressed in *E. coli* by Ranjan et al. (2015). They reported that rSt-Phy was produced in LB medium containing glycerol and glucose with a specific activity of 8000 U/mg total intracellular protein. The supplementation of rSt-Phy to dough had been found to be useful in the dephytinization of tandoori, naan and bread, as well as to increase the amount of inorganic phosphate and reduce the amount of sugars that are present. According to Ranjan and Satyanarayana (2016), in the expression of the codon-optimized phytase gene of *S. thermophile* (*St-Phy*) in *P. pastoris*, the recombinant *P. pastoris* harboring of phytase gene (*rSt-Phy*) secreted a 40-fold higher amount of phytase than the native fungal strain. Subsequently, the expression of codon-optimized *S. thermophile* (*rSt-Phy*) was used to investigate the glyceraldehyde phosphate dehydrogenase (GAP) promoter in *P. pastoris* (Maurya et al., 2017). They reported a result of about a 41-fold improvement in *rSt-Phy* production over the wild type strain. Recently, Mehmood et al. (2019) improved *S. thermophile* strain ST20 using physical and chemical mutagens for enhanced phytase activity. They used gamma rays and EMS (Ethyl Methane Sulfonate) mutagenesis to enhance the activity of phytase, for which the phytase activity was improved to 387 U/mL at 45°C. In addition, they also reported that the mutants produced through EMS displayed greater potential of phytase production when compared to the parent strain. The developing and improving production of heterologous proteins was determined under constitutive and inducible promoters’ systems (Parashar and Satyanarayana, 2016; Kluge et al., 2018). The expression of phytase genes phyA and appA2 were expressed in *P. pastoris* (constitutive or inducible) and *Saccharomyces cerevisiae* (inducible) by Lei and Kim (2005). The pGAPZαA vector and PPICZαA vector were used in the constitutive and inducible expressions for *P. pastoris*. To obtain the inducible expression in *S. cerevisiae*, the pYES2 vector was used. Subsequently, in 2017, the production of recombinant acidic phytase was enhanced in *P. pastoris* under dual promoters of constitutive (AOX) and inducible (Phy-GAP-AOX) conditions that were generated by Maurya. They found that it led to a 1.3-fold improvement in phytase production in mixed fed-batch cultivation when compared to that of Phy-AOX. Consequently, it was suggested that the improvement of the recombinant phytase gene could be beneficial to a number of production processes including the animal feed industry and the commercial bread baking industry. Notably, it can also be of benefit in deriving haloperoxidase and in plant growth-promoting.

## Crystal Structure and Protein Engineering of Fungal Phytases

**FIGURE 3:**
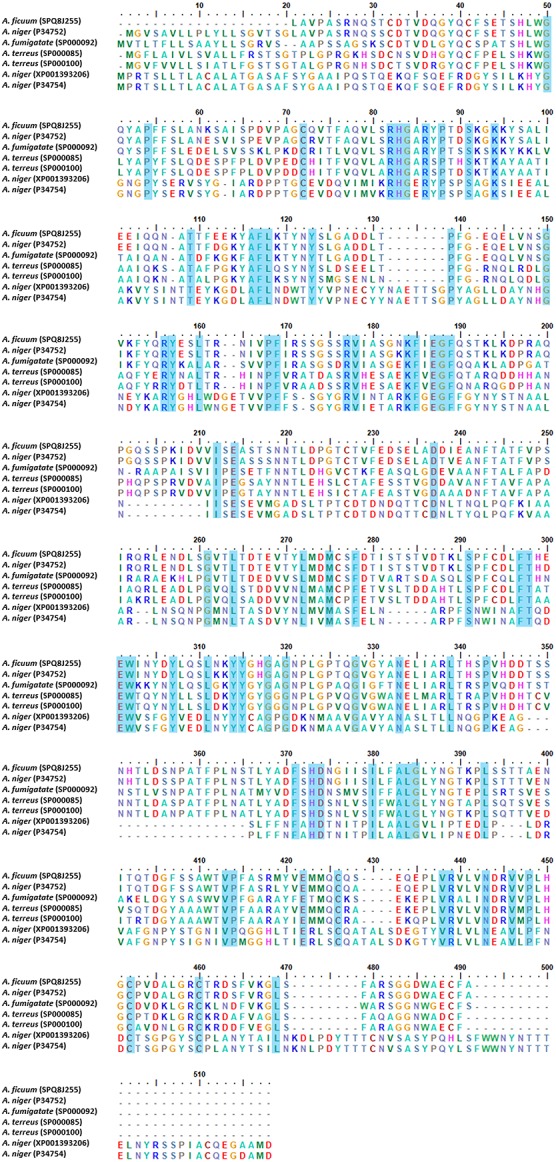
Multiple alignments of histidine acid phytase (HAPs) from genus *Aspergillus*. The alignments were performed using CLUSTAL_W.

The crystal structure analysis of phytases derived from bacteria, yeast, fungi, and plants has been reported by several researchers in terms of the distinct fold and biophysical properties that rationalized their structure (Yao et al., 2011). Phytases are classified into four groups according to the relevant catalytic mechanism; [(1) histidine acid phytases (HAPs), (2) β-propeller phytases (BPPs), (3) cysteine phytases (CPs), and (4) purple acid phosphatases (PAPs)] (Dailin et al., 2019). In the case of fungi, few phytase crystal structures from the genus *Aspergillus* have been studied. The studied *Aspergillus* species are namely; *A. niger* (Oakley, 2010), *A. ficuum*, *A. neoniger*, and *A. fumigatus* (Kostrewa et al., 1997; Liu et al., 2004; Xiang et al., 2004). Most fungal acidic phytases belong to HAPs and can be divided into two groups based on the optimum pH value of fungal HAPs, and their sub-classification in type A and B (Bei et al., 2009), PhyA (high specific activity for phytic acid and alkaline), and PhyB (acidic, low specific activity for phytic acid). Furthermore, PhyA is monomeric and PhyB is tetrameric (Wyss et al., 1998). The crystal structure of HAPs revealed three distinct domains; a large α-helical domain, a β-sheet domain, and a small α-helical domain (Dailin et al., 2019). The HAPs are composed of a large α/β-domain with a six-stranded β-sheet surrounded by several α-helices and a small α-domain. The HAPs structure also consists of *N*-acetylglucosamine residues and disulfide bonds. All 10 cysteine residues are involved in five disulfide bridges, but the disulfide bridge positions and the *N*-acetylglucosamine numbers are different in the *Aspergillus* species. Notably, the protein signature of HAPs is represented by the sequence consensus pattern [LIVM]-X-X-[LIVMA]-X-X-[LIVM]-X-R-H-[GN]-X-R-X-[PAS] (Liu et al., 2004^1^). Additionally, the protein sequences of HAPs type A and B obtained from the genus *Aspergillus* were aligned using CLUSTAL_W and were found to share a conserved active-site motif RHGX1RX2P (Figure 3). PhyA presents an active-site as RHGARYP. With regard to the catalytic importance of amino acid, histidine residue has been reported as a nucleophile in the formation of covalent phosphoenzyme intermediates (McTigue and Van Etten, 1978; Van Etten, 1982). The mechanism of acid phytase in the complex with inorganic phosphate revealed that two phosphates and four calcium ions were bound at the active site. The inorganic phosphate was then subsequently hydrolyzed by an activated water molecule. Finally, the hydrolyzed products amounted to *myo*-inositol, inositol, and inorganic phosphates (Zhang, 1998; Shin et al., 2001).

Phytase B obtained from *A. niger* (HAP) was comprised of 460 amino acid residues and contained five disulfide bonds at positions 52–368, 109–453, 197–422, 206–279, and 394–402, most of which were located in loops next to the surface (Kumar et al., 2013). In any case, *A. fumigatus* phytase consisted of 435 amino acid residues, six *N*-acetylglucosamine molecules, and five disulfide bonds that were present in the structure at positions 8–17, 48–391, 192–442, 241–259 and 413–421 (Xiang et al., 2004). Oakley (2010) presented a structural phytase model of phytase A obtained from *A. niger* that consisted of an α/β-domain, an α-domain, and an N-terminal extension. *N*-acetylglucosamine residues are bound to four sites of the phytase structure (N82, N184, N316, and N353) within the active site (Oakley, 2010). However, thermostability engineering of phytases is of interest for industrial and pharmaceutical applications. Site-directed mutagenesis, random mutation, molecular dynamic simulation, and protein glycosylation are methods of structural modification that are commonly employed when disulfide bonds, hydrogen bonds, ionic interaction and N/O-linked glycosylation are introduced in the phytases. Notably, this effectively improved their thermostability characteristics (Mullaney et al., 2010; Vasudevan et al., 2019). Mullaney et al. (2010) removed disulfide bridge site-directed mutagenesis number 2 from *A. ficuum* phytase, and this resulted in a complete loss of activity. Moreover, hydrogen bonds and ionic interaction can also support a degree of thermostability in phytases. For example, *A. fumigatus* phytase is heat resilient as it has a hydrogen bonding network in the E35 to S42 regions and in ionic interactions between R168 and D161 and R248 and D244. In another study, the Mn (2′-deoxyinosine 5′-triphosphate) random mutation method used on a protease-resistant phytase gene of *Penicillium* sp. developed two mutants with improved thermal stability and optimal temperature tolerance (Zhao et al., 2010). Formerly, using the molecular dynamic simulation, *A. niger* PhyA and its thermostable mutant possessing a 20% greater level of thermostability, were compared by evaluating the atomic root mean square deviation, the radius of gyration, and the number of hydrogen bonds and salt bridges that were present (Noorbatcha et al., 2013). Protein glycosylation is one of the most common structural modifications employed by biological systems to expand proteome diversity. Glycosylation in *A. niger* and *A. japonicus* phytases has been identified for its functional expression and thermostability when expressed in yeast systems (Han and Lei, 1999).

## Applications and Benefits of Fungal Phytases

### Feed Supplements

Phytases are of great interest in biotechnological applications in terms of the processing and manufacturing of human and animal nutrition since they have the potential to improve the efficient use of phosphorus and to reduce phytate content in food production and animal feed (Greiner and Konietzny, 2006; Yao et al., 2011; Singh et al., 2016). Monogastric animals, such as swine, poultry, and fish, lack or contain low levels of gastrointestinal phytases and they are unable to utilize the phytate phosphorus that is present in sources of food and animal feed. Therefore, they need inorganic phosphate supplements to meet their nutritional and growth-related needs, which in turn increases feed costs and levels of phosphorus pollution (Naves et al., 2012; Dersjant-Li et al., 2015). Phytase plays an important role in the animal feed industry because it enhances the digestion and absorption of phosphorus and certain other poorly available nutrients in monogastric diet supplements (Munir and Maqsood, 2013; Vasudevan et al., 2017). Various microorganisms are favored sources for industrial enzymes due to the ease of use that is associated with them, along with their cost-effectiveness, fast growth-rate and consistent production levels (Singh et al., 2016; Raveendran et al., 2018). Phytases produced by microorganisms are commonly used as a commercial feed additive. In 1999, the first generation of the fungal phytase obtained from *A. niger* was made commercially available in markets. At which point, phytases were further developed and became more widely commercially available (Lei et al., 2013; Singh et al., 2016). Several studies have investigated the applications of phytases as a feed additive.

#### Poultry

In broilers, the pretreatment of phytases in the digestive system of animals was investigated by feeding them a soybean meal (SBM) diet using *A. niger* phytase supplementation (Nelson et al., 1968). Nelson et al. (1968) studied the effects of phytase by pretreating corn–soya diets for broilers and reported that the availability of phosphorus increased by 60% when microbial phytase was given to broilers fed low phosphorus diets, while phosphorus concentrations in the chicken manure decreased by 50%. The reports also indicated that the bodyweights of male (13.2%) and female (5.8%) chickens increased after 21 days of phytase supplementation. Simons et al. (1990) and Zyla et al. (2001) reported that the addition of phytase in dietary phosphorus could decrease phosphorus levels in manure and increase body weight. Several studies have been carried out to determine the effect of microbial phytase on poultry growth. Englmaierova et al. (2015) evaluated the effect of different amounts of *A. niger* phytase on egg quality, along with the calcium and phosphorus digestibility of the hens. The results revealed the highest degree of eggshell percentage in terms of thickness on the index when 350 FTU/kg was applied. Kim et al. (2017) studied the effect of super-dosing phytase on the productive performance and egg quality in laying hens. They reported that the super-posing level of 20,000 FTU/kg phytase in diets had a positive effect on the egg production rate, but had no beneficial effect on egg quality in laying hens. Woyengo and Wilson (2019) reported that the supplementation of phytase at super-dose levels (≥2500 FTU/kg) had a more positive effect with regard to improving the ileal digestibility of energy, protein, P in maize and other forms of nutrient utilization in the modified diets prepared for poultry when compared with the supplementation of phytase administered at the industry recommended level (1,000 FTU/kg). Calcium (Ca) and phosphorus (P) are important nutrients for bone development and the metabolic processes involved with the enzyme cofactors present in poultry diet formulations (Menezes-Blackburn et al., 2015; Li et al., 2017). However, the concentration levels and rations for poultry must be close to their specified requirements. Consequently, a study on the effect of reducing dietary Ca levels and calcium, along with available phosphorus (Ca:aP) ratios in combination supplemented fungal phytases on poultry growth performance, nutrient digestibility, bone ash, and mineralization, was conducted (Delezie et al., 2015; de Souza Nardelli et al., 2018; Ajith et al., 2019).

#### Pigs

In pigs, the main active site for microbial phytase is in the stomach and upper part of the small intestine, a circumstance that is similar to poultry. Most of the phytases given to pigs are used to improve dietary phytate-P utilization and to improve their mineral and nutrient digestibility (Humer et al., 2015). The site of supplemental phytase activity in the gastrointestinal tract of young pigs was investigated by Yi and Kornegay (1996). They determined that supplemented *A*. *niger* fungal phytase in pig diets revealed that the digesta of the stomachs of pigs showed higher phytase activity than the digesta of the upper small intestine. The phytase activity levels in the stomach, as well as in the upper and lower parts of the small intestine, were 579, 348, and 53 FTU/kg, respectively when pig feed was supplemented with 1050 FTU/kg microbial phytases. Seynaeve et al. (2000) reported that supplementation of 500 FTU/kg *A. niger* phytase reduced intestine tract ileal digesta and total P (*P* = 0.09) and IP6-P (*P* < 0.05) values when compared with the non-treatment group. The supplementation of fungal phytase can reduce total P as well as inorganic *P*-values in feces and also improve overall growth performance and nutrient digestibility (Seynaeve et al., 2000; Dersjant-Li et al., 2015; McCormick et al., 2017).

#### Fish

Fungal phytase is not only used in the poultry and pig raising industries, but also in fisheries. Several studies on phytase supplementation in fish feed have involved different fish species (Kumar et al., 2012; Lemos and Tacon, 2017). For example, Yan et al. (2002) studied the effects of phytases at levels from 0 to 8,000 FTU/kg (*A. niger* phytase) in channel catfish (*Ictalurus punctatus*). They reported that the total phytate content in the stomachs of channel catfish was related to phytase inclusion levels. After feeding channel catfish for 2 h, total phytase content in the stomachs of fish fed with the phytase-supplemented feed was recorded at 92, 68, 50, 9, and 6% and at 500, 1000, 2000, 4000, and 8000 FTU/kg, respectively. A study by Forster et al. (1999) involving phytase-supplemented feed given to rainbow trout *Oncorhynchus mykiss* reported on the potential for using dietary phytase to improve the nutritive value of canola protein concentrate. Supplementation of phytase in fish feed indicated that dietary phytase improves the nutria value as well as increases the concentration levels of minerals in the plasma, bone, and the entire body. Additionally, it was also found to decrease the level of phosphorus that is discharged into aquatic environments.

### Food Additives

In addition to feeding additives, phytic acid is highly present in the flour and wholemeal flour of various types of dough and bread; therefore, phytases have been used as a food additive in fermentation processes and in various applications in the breadmaking process. For example, *A. ficuum* phytase has been used in legume dephosphorylation processes. It was reported that after mixing and incubating soybean meal with fungal phytase for 15 h, up to 78% of phytate was removed (Han and Wilfred, 1988). Turk and Sandberg (1992) studied the effects of phytase obtained from *A. niger* in InsP6 degradation during the breadmaking process. They reported that phytase preparation from *A. niger* used for making dough resulted in increased degradation of phytates. Later, the application of phytases in bread-making was also studied by Haros et al. (2001). Experiments have been performed by adding different levels of fungal phytase in whole wheat bread during the breadmaking process. Their results showed that specific bread volume increased, while crumb texture improved. Furthermore, Rosell et al. (2009) investigated the effects of different breadmaking processes, such as conventional, frozen dough, and frozen partially baked bread, and the effect of the storage period on the technological quality of fresh wholemeal wheat bread by adding *A. niger* phytase. They reported that the fungal phytase addition could be used in the breadmaking process and in the frozen storage of bread to overcome the detrimental effects of bran on the mineral bioavailability.

### Applications in Plant Growth Promotion

Phosphorus (P) is a major and critical component of cells and is a constituent in energy metabolism, and the biosynthesis of acids and cell membranes. It is also an important macronutrient for plant growth and development (Singh and Satyanarayana, 2011). Phosphorus deficiency in soil is a major problem for agricultural producers worldwide. Most soils contain significant amounts of total soil P that occurs in either an organic or inorganic form. A phytic acid is a major form of organic phosphorus in the soil, representing total organic phosphorus content between 10 and 50% (Mullen, 2005). Moreover, it is not readily available to plants as a source of phosphorus because it forms a complex with cations or adsorbs to various soil components. Therefore, the improvement of phosphorus nutrition requires the mobilization of organic and inorganic phosphorus (Richardson et al., 2001). Phosphate solubilizing microorganisms are ubiquitous in soil and can play an important role in the phosphorus cycle in nature as to serve as a readily available source of carbon and energy for their growth and reproduction (Whipps, 1990). In the rhizosphere, plant growth-promoting fungi (PGPF) solubilize or mineralize phosphorus and increase its availability to plants (Zhang et al., 2016; Hossain et al., 2017). Thakur et al. (2017) isolated and characterized extracellular phytase-producing *A. fumigatus* from the rhizospheric zone of maize fields. Phytase-producing fungi in the rhizosphere have been isolated and studied for their important role in promoting plant growth. *Aspergillus* was isolated as phytase-producing rhizofungi, and they were found to significantly improve the growth and phosphorus nutrition of *Arabidopsis* plants (Richardson et al., 2001). Furthermore, various *Aspergillus* species, such as *A. flavus*, *A. fumigatus*, and *A. rugulosus*, were used to promote the growth of plants (Tarafdar and Rao, 1996; Tarafdar and Gharu, 2006; Gaind and Singh, 2015). Gaind and Nain (2015) isolated various phytate-mineralizing fungi (PMF) and phosphatase-solubilizing fungi (PSF) from the rhizosphere soil of leguminous, cereal, and vegetable crops that belong to *Aspergillus*, *Trichoderma*, and *Penicillium*. They reported that *Penicillium chrysogenum* solubilizes the organic form of phosphorus and improves the available P in the soil while increasing the level of extractable organic P under alkaline soil conditions to benefit P nutrition. Singh and Satyanarayana (2010) investigated the role of phytase-producing fungi by increasing phosphorus content and promoting the growth of wheat (*Triticum aestivum* L.) seedlings. Tarafdar and Gharu (2006) also tested the significant role of the phytase producing fungus, *Chaetomium globosum*, for the improvement in plant biomass, root length, plant P concentration levels, seed and straw yields and seed P contents in wheat and pearl millet crops. According to the findings of a range of studies, it can be concluded that fungal phytase could be used to promote the growth of crop plants and to improve overall productivity levels.

### Applications in Therapeutics

In many parts of the world, humans consume plant-based food products as the main source of raw material food. Plant-based food products compost very important sources of nutrients (carbohydrates, protein, dietary fiber, and vitamins) and non-nutrients (Katina et al., 2005). Phytate is the primary storage compound of phosphorus in plant seeds and grains accounting for up to 90% of the total seed phosphorus (Loewus, 2002). It forms complexes with dietary minerals such as zinc, iron, calcium, magnesium, manganese, and copper, and causes mineral-related deficiency in humans (Lopez et al., 2002; Konietzny and Greiner, 2003). For instance, negative effects on mineral uptake, protein digestibility, carbohydrate, and lipid utilization have been recorded. In spite of the fact that phytates present a number of negative effects on human health, some positive effects have also been reported. Their consumption acts as an anticancer property by interrupting cellular signal transduction and cell cycle inhibition, and by enhancing natural killer cell activity (Kumar et al., 2010). The phytate substrates have been reported in various biomedical and biotechnological applications including those associated with antioxidant properties, as well as being identified as an anticancer agent, and a chelator with neuroprotective properties that can induce autophagy and reduce inflammation (Irshad et al., 2017). In addition, they have also displayed various therapeutic properties as anticancer agents (against colon cancer, breast cancer, hepatocellular carcinoma, prostate cancer, rhabdomyosarcoma, pancreatic cancer, and blood/bone marrow cancer) and have been found to be effective against Parkinson’s disease (Kumar et al., 2010; Irshad et al., 2017).

### Commercial Phytase Products

Phytases are beneficial enzymes for animal nutrition. They held the highest revenue share of 83.6% of the total industry in 2015^2^ and account for annual sales of US$ 350 million. About 70% of monogastric animal feed is supplemented with phytases (Ranjan and Satyanarayana, 2016). The first commercial phytase product was derived from *A. niger* and was classified as a 3-phytase. It was used in animal feed and was first marketed in 1999 under the trade name Natuphos. It was manufactured by Gist-Brocades (now DSM) and sold by BASF, Ludwigshafen, Germany. Later, the commercial product (Ronozyme^®^ P) belonging to a 6-phytase was derived from *Peniophora lycii*. Subsequently, a few fungal phytase products have been produced and marketed by other companies over the years (Table 5). On a commercial scale, phytase production is either carried out using phytate-producing fungi or recombinant DNA technology. The commercial products of Allzyme^®^ SSF and Adisseo were produced by naturally secreted enzymes that are synergistic with phytase. However, most fungal phytases used on a commercial scale were derived by using recombinant DNA technology. These commercial products are produced by recombinant filamentous fungal strains (Table 5).

**TABLE 5:** Common commercial phytases and fungal strains used.

Product	Company	Phytase source	Fungal strain used	References
Allzyme^®^ SSF	Alltech	*Aspergillus niger*	*Aspergillus niger* Non-recombinant	Lei et al., 2013
Finase^®^ P/L	AB Vista	*Aspergillus niger* PhyB	*Trichoderma reesei*	Simon and Igbasan, 2002; Misset and Whitaker, 2003; European Union, 2004; Haefner et al., 2005
Natuphos^®^	BASF	*Aspergillus niger* PhyA	*Aspergillus niger*	Simon and Igbasan, 2002; Misset and Whitaker, 2003; European Union, 2004; Haefner et al., 2005
Ronozyme^®^ P	Novozyme/DSM	*Peniophora lycii* PhyB	*Aspergillus oryzae*	Simon and Igbasan, 2002; European Commission, 2004; European Union, 2004; Haefner et al., 2005
Rovabio	Adisseo	*Penicillium funiculosum*	*Penicillium funiculosum* Non-recombinant	Greiner and Konietzny, 2012

Several commercial phytase products are used as supplements for monogastric animal feeds. The function of phytase in animal feeds and digestive systems is critically important. In addition, different phytases used for animal feed applications differ in their enzymatic properties. For instance, the optimum pH and temperature values of Ronozyme^®^ P were 4–4.5 and 50–55°C, respectively (Lassen et al., 2001). While Natuphos^®^ revealed optimum pH and temperature values at 2.0, 5–5.5, and 65°C, respectively (Wyss et al., 1999; Zhang et al., 2007; Weaver et al., 2009). The performance of commercial phytases was also determined in terms of their enzymatic properties under identical assay conditions. For example, the commercial product named Rovabio was used to investigate growth performance and intestinal viscosity in broiler chicks fed (Lee et al., 2010). Additionally, Ronozyme HiPhos was used to investigate the apparent ileal digestibility of minerals and amino acids in ileorectal anastomosed pigs (Guggenbuhl et al., 2012). Menezes-Blackburn et al. (2015) used Natuphos^®^ and Ronozyme^®^ P in a study involving *in vitro* stimulation of the digestive tracts of poultry.

## Conclusion and Future Perspectives

Fungal phytases have gained increasing amounts of interest for use in food production and in the feed industries as a way of improving nutrition quality and reducing levels of phosphorus pollution. The study of different biological properties of fungal phytase is important and can assist researchers in improving the levels of phytase activity and stability for nutritional and industrial uses. However, only a few fungal strains have been studied in terms of phytase production. Therefore, the discovery of new fungal species with advanced phytase properties and levels of stability will be necessary. In addition, the cloning and protein engineering of potential phytase producing fungal species will also be extremely advantageous.

## Author Contributions

KJ, NS, JK, and SL designed the general plan of the review. KJ conducted the necessary literature searches, created figures, and wrote the manuscript. PK and WP conducted a literature search and wrote the manuscript. WP conducted a bioinformatic analysis of the protein sequences. All authors read, revised and approved the final manuscript.

## Conflict of Interest

The authors declare that the research was conducted in the absence of any commercial or financial relationships that could be construed as a potential conflict of interest.

## Footnotes

1 http://www.expasy.ch/cgi-bin/get-prodoc-entry?PDOC00538

2 https://www.grandviewresearch.com
